# Leadership cycles, styles, and antecedent factors: the perspective of coaches and young soccer athletes from national Slovak leagues

**DOI:** 10.3389/fpsyg.2023.1218290

**Published:** 2023-08-07

**Authors:** Elena Lisá, Jacinta Sousa, Catarina Morais, António Rui Gomes

**Affiliations:** ^1^Institute of Applied Psychology, Faculty of Social and Economic Sciences, Comenius University in Bratislava, Bratislava, Slovakia; ^2^Adaptation, Performance, and Human Development Research Group, Psychology Research Centre, University of Minho, Braga, Portugal; ^3^Faculty of Education and Psychology, Research Centre for Human Development, Universidade Católica Portuguesa, Porto, Portugal; ^4^Psychology Research Centre, University of Minho, Braga, Portugal

**Keywords:** leadership efficacy model, transformational leadership, leadership philosophy, young athletes, football coaches

## Abstract

**Introduction:**

Building positive relationships and interactions between coaches and athletes is critical to an athlete’s success. The current study aimed to overview how coaches and their young athletes perceive three elements of the Leadership Efficacy Model (philosophy, practice, and criteria). The aim was examined with four goals of analysis: the perceptions of coaches and athletes about coaches’ leadership philosophy, practice, and criteria (1); the differences between athletes’ and coaches’ perceptions of leadership cycles (2); the differences between athletes’ and coaches’ perceptions of leadership styles (3); and the differences between athletes’ and coaches’ perceptions of leadership antecedent factors (4).

**Methods:**

The study involved 304 athletes and 20 coaches competing in the youth national leagues U15, U16, U17, and U19. Two-source data collection was applied: coaches completed the questionnaires from their point of view, and so did athletes. The coaches were paired then with their athletes to compare the answers. Coaches fulfilled Leadership Cycles Questionnaire (LEQ), Multidimensional Scale of Leadership in Sport (MSLS), and Leadership Antecedent Factors Questionnaire (LAFQ). Athletes completed the same questionnaires as the coaches did and also fulfilled the Sport Performance Perception Questionnaire (SPPQ). Athletes’ age and SPPQ served as control variables.

**Results:**

Both athletes (37.5%) and coaches (40%) perceived that the philosophy of the leadership efficacy model should be increased. Coaches evaluated their philosophy (*F* = 4.43; *p* = 0.036; *η^2^* = 0.014), support in MSLS (*F* = 5.05; *p* = 0.025; *η^2^* = 0.016) and active management in MSLS (*F* = 4.08; *p* = 0.044; *η^2^* = 0.013) higher than their athletes. The athletes assessed the maturity of the team members (LAFQ dimension) (*F* = 13.98; *p* <0.001; *η^2^* = 0.044), negative feedback in MSLS (*F* = 6.02; *p* = 0.015; *η^2^* = 0.020), and passive management in MSLS (*F* = 4.95; *p* = 0.027; *η^2^* = 0.016) higher than their coaches.

**Discussion:**

The tendency of coaches to have a more positive perception of their leadership behavior compared to their athletes represents the coach-athlete perception gap of leadership. Future research can examine the efficacy of congruent perceptions of leadership between athletes and coaches during the sports season and the impact produced by objective performance indicators.

## 1. Introduction

Establishing positive relationships and interaction between coaches and athletes is vital for athletes’ success (Park and Seo, 2019). It is difficult to define the number of factors that can reinforce the relationship between coaches and athletes and promote sports success. Leadership style (de Albuquerque et al., 2021), particularly transformational leadership (Álvarez et al., 2019), coaching philosophy (Collins, 2021; Subagyo, 2023), and the conditions for leadership (Bormann et al., 2016; Reardon et al., 2022; Warmath et al., 2022) are among the most important. Leadership styles play a significant role in the way coaches influence athletes and stimulate their sports success, as is the case of the transformational leadership style (Álvarez et al., 2019; McGuckin et al., 2022), democratic decision-making style (Kim et al., 2021), and social support directed to reinforce personal trust and confidence with athletes (Burns et al., 2022).

Recent research confirms the relevance and importance of coaches’ leadership (Park and Seo, 2019; de Albuquerque et al., 2021) for the athletes’ outcomes and interpersonal relationships with athletes. For example, perceived transformational leadership in athletes increases the perception of a coach’s effectiveness through task climate (Álvarez et al., 2019). Athletes respond in a positive way when coaches laugh and support their careers. They value strong personal relationships with coaches in the same they value the technical influence of coaches, perceiving well-being and athletic performance as equally important (Burns et al., 2022). When the athletes evaluate their coach as a democratic leader, they have better personal and social development (de Albuquerque et al., 2021). Young athletes develop a relationship with their coach and a sense of belonging when they perceive their coach as an affective and motivating one. On the contrary, when they perceive their coach as autocratic, they do not develop their personal and social skills and goal setting. Therefore, teaching techniques and leadership styles are important to the coach’s leadership profile.

Another important factor that contributes to coach efficacy and positive relationships with athletes is coaching philosophy (Gould et al., 2017; Collins, 2021). This concept has been stated as the values, beliefs, or principles expressed by coaches about their activity (Vallée and Bloom, 2016), and it influences how coaches prepare athletes for competition. The philosophy of coaches (particularly those with successful careers) has generated the interest of researchers. However, it is still unclear how coaching philosophy manifests itself in coaches’ everyday interactions with athletes (Callary et al., 2013; Gomes et al., 2018).

The last factor influencing the coach-athlete relationship depends on the conditions under which the leadership process occurs. How athletes perceive the leadership style of their coaches is affected by time in the sports program (de Albuquerque et al., 2021), household income (Warmath et al., 2022), their individual characteristics (Bormann et al., 2016) or the season outcome (Reardon et al., 2022). The recognized antecedent factors of leadership as intervening or moderating variables are conditions of the coach/leader’s characteristics (goals, beliefs, values, psychological resources, sex, age, and personality), team members’ characteristics (goals, beliefs, values, psychological resources, sex, age, and personality) and situational characteristics (expectations/organizational goals, hierarchical level and power, values, and norms) (Gomes, 2020; Gomes et al., 2022). They all seem to be essential factors for understanding how coaches act and establish positive relationships with athletes on a daily basis.

The coaches’ philosophy, leadership style, and conditions are three crucial factors for explaining and understanding the quality of the coach-athlete relationship. However, research tends to study these factors in isolation. Because of that, this study aims to analyze these factors as interactive parts of the Leadership Efficacy Model (Gomes, 2020). This model identifies the leadership cycles (philosophy, practice, and criteria) as central elements of leadership efficacy, proposing that they should be linearly congruent (Gomes et al., 2022). In this way, the model sustains that leadership efficacy increases when leaders/coaches assume linear relations between philosophy (ideas that support the coaching activity), practice (behaviors supposed to fulfill the philosophy), and criteria (indicators to monitor the ideas and behaviors of leadership) (Gomes, 2020). Also important, the model indicates that leadership styles (i.e., transformational leadership, transactional leadership, and decision-making leadership) and antecedent factors of leadership (leader, follower, and situational characteristics) can influence the impact produced by leadership cycles in leadership efficacy (Gomes et al., 2022). According to the Leadership Efficacy Model, when leaders implement the leadership cycles by using positive leadership styles and considering antecedent factors of leadership, they can increase the impact of the leadership cycles in the final leadership efficacy (measured, for example, in terms of athletes’ psychological experiences in sports or sport performance) (Gomes et al., 2022). For the case of leadership styles, it is proposed that the “optimal profile of leadership” occurs when leaders use mainly transformational leadership behaviors, higher levels of positive feedback and lower levels of negative feedback from transactional leadership, and when they use higher levels of active management and lower levels of management from decision-making leadership (Gomes, 2020). For the antecedent factors of leadership, it is proposed that leaders consider the influence of three factors in their leadership cycles: leaders, team members, and situational characteristics. In practical terms and applying to sports contexts, coaches should adjust their leadership plan to their and athletes’ personal and professional characteristics and situational conditions (Gomes, 2020) because the congruence between the perception of athletes and coaches is crucial, as shown in the research on athletes’ self-efficacy (Stephen et al., 2022).

Due to the recent formulation of the model (Gomes, 2020), there are not many research indications of its utility, particularly in the case of youth sports. For example, Gomes and Resende (2014), in a study with soccer and futsal athletes from main divisions, concluded that the optimal profile of leadership (particularly the transformational leadership) was important for explaining satisfaction with leadership (61% of variance explained) and coach-athlete compatibility (50% of variance explained). In the same way, Gomes et al. (2020) in a study with soccer athletes, found that the optimal profile of leadership (e.g., transformational leadership, positive feedback from transactional leadership, and active management from decision-making leadership) was higher reported by athletes with higher perceptions of individual goal achievement than by athletes who reported lower individual goal achievement. More recently, Gomes et al. (2022) concluded that the higher congruence in leadership cycles perceived by soccer athletes corresponded to higher perceptions of team performance and that optimal leadership profiles and higher leadership favorability corresponded to higher individual and team performance. Although the interest of these studies, the importance of analyzing this model in youth sports is due to the vital role of coaches in shaping young athletes’ experiences and outcomes in sports. For example, one crucial area of inquiry is the examination of different leadership styles and their impact on athletes’ experiences. Some studies have shown that coaches who adopt a transformational leadership style are more likely to foster positive athlete outcomes such as intrinsic motivation and psychological well-being (Hodge et al., 2009). However, coaches who exhibit controlling behaviors or are overly critical can undermine athletes’ motivation and lead to adverse outcomes such as burnout (Mageau and Vallerand, 2003; Reinboth and Duda, 2006). Studying coaches’ leadership in youth sports is critical because it helps identify the coaching behaviors and practices that most effectively promote positive athlete outcomes and experiences. By better understanding the role of coaches in youth sports, we can support the development of coaches to be better equipped to provide a positive and supportive environment for young athletes.

The current study aims to provide an overview of how three elements of the Leadership Efficacy Model (leadership cycles, leadership styles, and antecedent factors of leadership) are perceived by both coaches and their young athletes. Four objectives were formulated: (1) To describe the perceptions of coaches and athletes about coaches’ leadership philosophy, practice, and criteria (leadership cycles). (2) To analyze the differences between athletes’ and coaches’ perceptions of leadership cycles, controlling for athletes’ age and perceptions of sport performance. (3) To analyze the differences between athletes’ and coaches’ perceptions of leadership styles, controlling for athletes’ age and perceptions of sport performance. (4) To analyze the differences between athletes’ and coaches’ perceptions of leadership antecedent factors, controlling for athletes’ age and perceptions of sport performance.

## 2. Methods

### 2.1. Participants

This study includes a convenience sample based on the following criteria: young football athletes who competed in the U15, U16, U17, or U19 national Slovak leagues and their respective coaches. The study involved 304 athletes from 20 different teams, competing in U15 (1%), U16 (23%), U17 (40%), and U19 (36%) national leagues. Participants were all male, aged between 15 and 18 years old (*M* = 16.42; SD = 1.00), and had between 3 and 15 years of sports practice (*M* = 9.96; SD = 1.92).

Furthermore, the study included 20 coaches (1 per team): 1 (5%) training U15, 4 (20%) training U16 teams, 8 (40%) training U17 athletes, and 7 (35%) in category U19. All coaches were male, between the ages of 24 and 54 (*M* = 37.25; SD = 8.69), and had between 5 and 20 years of experience in this sport (*M* = 10.65; SD = 3.96).

### 2.2. The research ethics

The study was approved by the institution’s Ethics Committee of the fourth author (SECSH 008/2016). Subsequently, the football clubs that met the inclusion criteria were contacted to obtain their permission to collect the data. After the clubs’ permission and before the data collection, informed consent was sent to all athletes and their legal tutors (if athletes were underage) that included all the necessary information about the study and the voluntary nature of their participation, as well as information regarding confidentiality and anonymity of all data collected. When informed consent was signed, the evaluation protocol was delivered to fulfill.

### 2.3. Eligibility criteria for research sample

The research team contacted the director of the youth football club in Slovakia. After agreeing to participate in the study, we sent the directors the informed consent forms. The directors delivered them to the coaches, who obtained informed consent from parents/athletes. After acquiring the consent, we delivered the link with the study questionnaires. The coaches could invite colleagues from other clubs to the research too, and they did it. The snowball method of data collection was applied. All coaches could leave their email addresses in an open question to get their team results. All participants could leave their email addresses to get their individual results. This was done to increase the active participation of the coaches in the study and their motivation to invite athletes. The individual or team reports were electronically sent to participants who demonstrated interest. However, agreement from the club director or the parents did not ensure that the athletes or coaches really participated in the study because the participation was voluntary and anonymous. We collected sample data from 439 athletes and 34 coaches, from which we excluded 135 athletes and 14 coaches, mainly for two reasons: (a) athletes and coaches that did not provide the information about the club they belong to (this was necessary for pairing them into the team with their coach); and (b) number of participants included in a team was very low (only one coach or one athlete in a team).

### 2.4. Measurements

#### 2.4.1. Leadership cycles questionnaire

The LCQ (Gomes et al., 2022; α_study_ = 0.97) evaluates the relationship between the leadership cycles (i.e., conceptual cycle and practical cycle), including three dimensions: (a) leadership philosophy: coaches’ principles, ideas, and values about what leadership is and what it is like to be a leader (5 items, α_study_ = 0.87); (b) leadership practice: daily behaviors assumed by coaches, to put into practice their philosophy (5 items, α_study_ = 0.88); and (c) leadership criteria: indicators used by coaches to evaluate their philosophy and practice of leadership (5 items, α_study_ = 0.90). The 15 items were answered twice: (1) current coaches’ behaviors: actual coaches’ behaviors; and (2) preferred coaches’ behaviors: ideal coaches’ behaviors. The items were presented on a five-point Likert scale (*1 = Never; 5 = Always*). The scores for each dimension were obtained by calculating their mean values. Subsequently, the differences between the preferred and current coaches’ behaviors resulted in the Leadership Cycles Congruence Index (LCCI). The LCCI results were transformed into a modular variable so that numbers closer to 0 indicated greater congruence between the actual and preferred coaches’ behaviors. With this final variable, the median (Md = 0.27) was used to divide athletes into two groups: High congruence (≤ 0.27) and low congruence (> 0.27). The confirmatory factorial analysis conducted for the current study revealed good psychometric properties of the instrument {χ2(87) = 308.043, *p* = < 0.001; χ2/df = 3.541; CFI = 0.950; TLI = 0.940; RMSEA = 0.077 [90% CI (0.068; 0.087), pclose = < 0.001]}.

#### 2.4.2. Multidimensional scale of leadership in sport

The MSLS (Gomes et al., 2021; α_study_ = 0.88) evaluates athletes’ perceptions of the coaches’ leadership behaviors in nine dimensions: (a) vision: establishment of a positive future for athletes (4 items, α_study_ = 0.80); (b) inspiration: actions that motivate athletes to do their tasks (4 items, α_study_ = 0.74); (c) instruction: indications of what can be done to improve athletes’ sport skills (4 items, αstudy = 0.74); (d) individualization: consideration of athletes’ personal needs and expectations (4 items, α_study_ = 0.79); (e) support: concern about athletes’ well-being (4 items, α_study_ = 0.76); (f) positive feedback: recognition of athletes’ good performance and efforts (4 items, α_study_ = 0.65); (g) negative feedback: punishment of athletes’ inadequate performance or behaviors (4 items, α_study_ = 0.61); (h) active management: behaviors of active decision making, involving athletes in the decision-making process (4 items, α_study_ = 0.68); and (i) passive management: actions of avoidance or delay in decision-making process (4 items, α_study_ = 0.69). The 36 items were answered on a five-point Likert scale (*1 = Never; 5 = Always*). To calculate the scores, both negative feedback and passive management were reversed. Subsequently, the scores of each dimension were obtained by calculating their mean values. Then, the Optimal Profile of Leadership Index (OPLI) was calculated based on the average of all dimensions. The median of the OPLI variable (Md = 3.89) was used to split the participants into two groups: Low OPLI (≤ 3.89) and high OPLI (> 3.89). The confirmatory factorial analysis revealed good psychometric properties of the instrument in the current study {χ2(558) = 1461.752, *p* = < 0.001; χ2/df = 2.620; CFI = 0.865; TLI = 0.848; RMSEA = 0.062 [90% CI (0.058; 0.065), pclose = < 0.001]}.

#### 2.4.3. Leadership antecedent factors questionnaire

The LAFQ (Gomes et al., 2022; α_study_ = 0.92) evaluates the antecedent factors that can influence leadership efficacy, including five dimensions: (a) leaders’ task orientation: leaders’ attention to the technical and productive aspects of work (3 items, α_study_ = 0.83); (b) leaders’ people orientation: leaders’ interest in the individual aspects of employees, such as expectations and values (3 items, α_study_ = 0.72); (c) team members’ technical maturity: team members capability and knowledge about the tasks and goals (3 items, α_study_ = 0.65); (d) team members’ psychological maturity: team members’ self-confidence and openness to take on the responsibility to carry out their tasks (3 items, α_study_ = 0.69); and (e) context: environmental factors that can impact leaders’ actions (3 items, α_study_ = 0.50). These five dimensions can be aggregated in three areas: (a) leader (includes leaders’ task and people orientations), (b) team members (includes team members’ technical and psychological maturities), and (c) context (the same presented before) that were used in this study. The 15 items were responded twice: (1) current situation: actual conditions that describe each antecedent factors; and (2) preferred situation: optimal conditions in each antecedent factors; items were presented on a five-point Likert scale (*1 = Never; 5 = Always*). The scores for each dimension were obtained by calculating their average. Then the differences between the preferred and current situation resulted in the Leadership Favourability Index (LFI). The LFI results were converted into a modular variable so that numbers closer to 0 indicated greater favorability. The median (Md = 0.22) of this final variable was used to divide the athletes into two groups: High favourability (≤ 0.22) and low favourability (> 0.22). The confirmatory factorial analysis revealed good psychometric properties of the instrument in the current study {χ2(80) = 239.051, *p* = < 0.001; χ2/df = 2.988; CFI = 0.925; TLI = 0.901; RMSEA = 0.068 [90% CI (0.058; 0.078), pclose = 0.001]}.

#### 2.4.4. Sport performance perception questionnaire

The SPPQ (Gomes et al., 2020; α_study_ = 0.93) evaluates the perception of individual performance (5 items, αstudy = 0.90) and team performance (5 items, α_study_ = 0.94) and was only responded to by the athletes. The ten items were presented on a five-point Likert scale (*1 = Completely disagree; 5 = Completely agree*), and the scores of both dimensions were obtained by calculating the average of participants’ responses. The confirmatory factorial analysis revealed good psychometric properties of the instrument in the current study: {χ2(26) = 59.456, *p* = < 0.001; χ2/df = 2.287; CFI = 0.990; TLI = 0.983; RMSEA = 0.055 [90% CI (0.036; 0.073), pclose = 0.308]}.

### 2.5. Procedures

Data collection was applied in a two-source data design (athletes and their coaches), to minimize the risk of common method bias (Rodríguez-Ardura and Meseguer-Artola, 2020). Participants completed the questionnaires anonymously in an online form during the Covid-19 pandemic. The researchers matched athletes to their coaches according to information about the club and the category. Each athlete was paired with their coach on the line in the data set. The athletes completed all the instruments, and the coaches completed the first three. Coaches did not meet the SPPQ because it is designed for the self-assessment of athletes (Gomes et al., 2020). The analyses used sport performance (SPPQ) as a control variable.

### 2.6. Data analysis

For the descriptive (frequencies) and inferential analysis (Pearson correlations, ANOVA, confirmatory factor analysis), the statistical software IBM SPSS (version 27.0) was used, which made it possible to respond to the previously established objectives. A frequency analysis of the non-modular LCCI variable was conducted to respond to the first objective for athletes and coaches. Then, repeated measures analysis was performed. The following statistical characteristics were applied: correlation coefficient (*r*), *F*-tests statistics (*F*), significance level (*p*), partial eta squared (*η*^2^), observed power (*π*), chi-square test (*χ*2), degrees of freedom (df), Comparative fit index (CFI); Tucker–Lewis index (TLI); Root mean square error of approximation (RMSEA). Age and SPPQ (individual and team performance) were established as covariates to respond to the remaining objectives. The win/lose season (Reardon et al., 2022) and perceiving team and individual performance affect the perception of the coaching style.

## 3. Results

### 3.1. Correlations between variables

Regarding the significant Pearson correlations coefficients (cf. Table 1), and starting with the LEQ variables, it was confirmed that both philosophy and criteria correlated negatively with Leadership Cycles Congruence Index (LCCI) (*r =* −0.264^***^; *r =* −0.179^**^) and passive management (*r =* −0.313^***^; *r =* −0.191^**^); practice correlated negatively with LCCI (*r =* −0.278^***^), passive management (*r =* −0.279^***^) and Leadership Favourability Index (LFI) (*r =* −0.131^*^); and the LCCI correlated negatively with all variables, excluding LFI – the negative correlation coefficients varied between −0.023 and − 0.188^***^. Secondly, regarding the MSLS, the variables vision and active management correlated negatively to LCCI (*r =* −0.131^*^; *r =* −0.119^*^) and LFI (*r =* −0.144^*^; *r =* −0.172^**^); inspiration, instruction, positive feedback, and Optimal Profile of Leadership Index (OPLI) correlated negatively to LCCI (*r =* −0.170^**^; *r =* −0.188^***^; *r =* −0.117^*^; *r =* −0.146^**^) and passive management (*r =* −0.214^***^; *r =* −0.323^***^; *r =* −0.129^*^; *r =* −0.335^***^); individualization correlated negatively to LCCI (*r =* −0.184^**^), passive management (*r =* −0.186^**^), and LFI (*r =* −0.127^*^); support correlated negatively to LFI (*r =* −0.115^*^); negative feedback correlated negatively to OPLI (*r =* −0.233^***^); and passive management correlated negatively to most variables, with exclusion to negative feedback and SPPQ. For the LAFQ variables, leader and context correlated negatively to passive management (*r =* −0.191^***^; *r =* −0.160^**^) and LFI (*r =* −0.285^***^; *r =* −0.279^***^); team correlated negatively to LFI (*r =* −0.280^***^); and LFI correlated negatively to all variables, excluding LCCI and SPPQ, with correlation coefficients between *r = 0*.27 and *r =* −0.285. Finally, the SPPQ variable correlated negatively to LFI (*r =* −0.137^*^).

**Table 1:** Pearson correlation coefficients (*r*) between variables of the study.

	1.	2.	3.	4.	5.	6.	7.	8.	9.	10.	11.	12.	13.	14.	15.	16.	17.	18.	19.
LEQ																			
1. Philosophy	–	–	–	–	–	–	–	–	–	–	–	–	–	–	–	–	–	–	–
2. Practice	0.774^***^	–	–	–	–	–	–	–	–	–	–	–	–	–	–	–	–	–	–
3. Criteria	0.753^***^	0.733^***^	–	–	–	–	–	–	–	–	–	–	–	–	–	–	–	–	–
4. LCCI	−0.264^***^	−0.278^***^	−0.179^**^	–	–	–	–	–	–	–	–	–	–	–	–	–	–	–	–
MSLS																			
5. Vision	0.476^***^	0.558^***^	0.410^***^	−0.131^*^	–	–	–	–	–	–	–	–	–	–	–	–	–	–	–
6. Inspiration	0.591^***^	0.691^***^	0.566^***^	−0.170^**^	0.632^***^	–	–	–	–	–	–	–	–	–	–	–	–	–	–
7. Instruction	0.690^***^	0.707^***^	0.645^***^	−0.188^***^	0.520^***^	0.745^***^	–	–	–	–	–	–	–	–	–	–	–	–	–
8. Individualization	0.598^***^	0.677^***^	0.564^***^	−0.184^**^	0.610^***^	0.624^***^	0.680^***^	–	–	–	–	–	–	–	–	–	–	–	–
9. Support	0.406^***^	0.472^***^	0.436^***^	−0.076	0.552^***^	0.444^***^	0.505^***^	0.631^***^	–	–	–	–	–	–	–	–	–	–	–
10. Positive Feedback	0.502^***^	0.553^***^	0.476^***^	−0.117^*^	0.545^***^	0.578^***^	0.584^***^	0.581^***^	0.443^***^	–	–	–	–	–	–	–	–	–	–
11. Negative Feedback	0.135^*^	0.032	0.091	−0.066	−0.060	0.059	−0.007	−0.109	−0.059	−0.052	–	–	–	–	–	–	–	–	–
12. Active Management	0.404^***^	0.432^***^	0.360^***^	−0.119^*^	0.544^***^	0.432^***^	0.456^***^	0.574^***^	0.569^***^	0.477^***^	0.019	–	–	–	–	–	–	–	–
13. Passive Management	−0.313^***^	−0.279^***^	−0.191^***^	−0.023	−0.061	−0.214^***^	−0.323^***^	−0.186^**^	0.031	−0.129^*^	0.146^*^	0.072	–	–	–	–	–	–	–
14. OPLI	0.645^***^	0.727^***^	0.594^***^	−0.146^**^	0.772^***^	0.766^***^	0.803^***^	0.842^***^	0.725^***^	0.729^***^	−0.233^***^	0.687^***^	−0.335^***^	–	–	–	–	–	–
LAFQ																			
15. Leader	0.591^***^	0.704^***^	0.566^***^	−0.070	0.456^***^	0.617^***^	0.662^***^	0.673^***^	0.479^***^	0.540^***^	0.034	0.492^***^	−0.191^***^	0.683^***^	–	–	–	–	–
16. Team	0.286^***^	0.321^***^	0.276^***^	0.082	0.332^***^	0.313^***^	0.346^***^	0.313^***^	0.265^***^	0.229^***^	−0.056	0.281^***^	−0.069	0.374^***^	0.404^***^	–	–	–	–
17. Context	0.474^***^	0.538^***^	0.458^***^	−0.035	0.335^***^	0.399^***^	0.449^***^	0.396^***^	0.343^***^	0.376^***^	−0.011	0.320^***^	−0.160^**^	0.470^***^	0.505^***^	0.399^***^	–	–	–
18. LFI	−0.042	−0.131^*^	0.027	0.134^*^	−0.144^*^	−0.074	−0.039	−0.127^*^	−0.115^*^	−0.084	0.010	−0.172^**^	−0.127^*^	−0.110	−0.285^***^	−0.280^***^	−0.279^***^	–	–
SPPQ																			
19. SPPQ	0.058	0.084	0.093	−0.002	0.137^*^	0.091	0.079	0.089	0.103	0.059	−0.087	0.085	0.084	0.109	0.086	0.186^**^	0.145^*^	−0.137^*^	–

^*^ < 0.050, ^**^ < 0.010, ^***^ < 0.001.

### 3.2. Leadership cycle of philosophy, practice, and criteria

Table 2 indicates the frequency and percentage of athletes’ and coaches’ perceptions regarding whether the coach should decrease, maintain, or increase the leadership philosophy, practice, or criteria. In this sense, it is possible to conclude that regarding the leadership philosophy, both athletes (37.5%) and coaches (40%) perceived that this behavior should be increased, meaning that coaches should make their ideas and principles more explicit. Regarding leadership practice (40.5% vs. 25%) and criteria (37.8% vs. 15%), athletes perceived that their coaches should increase their behaviors. On the contrary, coaches perceived that their behaviors on these dimensions were appropriate and should be maintained (37.5% vs. 25% and 36.5% vs. 75%). Thus, athletes perceived that their coaches should make more explicit the behaviors used to put into practice their philosophy, as well as the indicators used to evaluate their ideas and their implementation.

**Table 2:** Leadership cycles evaluation: coaches and athletes’ perspectives.

	Philosophy		Practice		Criteria	
	Athletes *n* (%)	Coaches *n* (%)	Athletes *n* (%)	Coaches *n* (%)	Athletes *n* (%)	Coaches *n* (%)
Decrease	77 (25.3%)	5 (25%)	67 (22%)	6 (30%)	78 (25.7%)	2 (10%)
Maintain	113 (37.2%)	7 (35%)	114 (37.5%)	9 (45%)	111 (36.5%)	15 (75%)
Increase	114 (37.5%)	8 (40%)	123 (40.5%)	5 (25%)	115 (37.8%)	3 (15%)

### 3.3. Leadership cycles, styles, and antecedent factors

The analysis presented in Table 3 identified the differences between athletes’ and coaches’ perceptions of leadership cycles, leadership styles, and antecedent leadership factors, with athletes’ age and perception of sport performance established as covariates. For each dimension, a repeated measure analysis was performed.

**Table 3:** Comparison athletes-coaches in leadership cycles, leadership styles, and leadership antecedent factors.

	Athletes	Coaches	*F* (1, 301)	*p*	ηρ2	π
	*M* (*SD*)	*M* (*SD*)				
LEQ						
Philosophy	4.31 (0.65)	4.64 (0.37)	4.43	0.036	0.014	0.55
Practice	4.23 (0.69)	4.73 (0.35)	1.38	0.241	0.005	0.22
Criteria	4.22 (0.69)	4.62 (0.44)	0.47	0.494	0.002	0.11
LCCI	0.08 (0.36)	−0.01 (0.14)	0.64	0.426	0.002	0.13
MSLS						
Vision	3.71 (0.77)	4.22 (0.58)	1.82	0.178	0.006	0.27
Inspiration	4.23 (0.63)	4.74 (0.25)	0.23	0.630	0.001	0.08
Instruction	4.35 (0.62)	4.79 (0.33)	1.61	0.206	0.005	0.24
Individualization	4.07 (0.65)	4.43 (0.42)	2.76	0.097	0.009	0.38
Support	3.59 (0.85)	4.39 (0.48)	5.05	0.025	0.016	0.61
Positive feedback	3.99 (0.62)	4.56 (0.29)	0.54	0.463	0.002	0.11
Negative feedback	2.98 (0.71)	2.97 (0.66)	6.02	0.015	0.020	0.69
Active management	3.41 (0.82)	3.65 (0.82)	4.08	0.044	0.013	0.52
Passive management	1.84 (0.80)	1.59 (0.54)	4.95	0.027	0.016	0.60
OPLI	3.84 (0.47)	4.25 (0.27)	2.03	0.155	0.007	0.29
LAFQ						
Leader	4.34 (0.57)	4.75 (0.31)	0.19	0.663	0.001	0.07
Team members	4.24 (0.55)	3.75 (0.39)	13.98	<0.001	0.044	0.96
Context	4.23 (0.59)	4.08 (0.75)	0.36	0.550	0.001	0.09
LFI	0.23 (0.33)	0.53 (0.51)	0.69	0.406	0.002	0.13

*F*-tests statistics (*F*), significance level (*p*), partial eta squared ( ηρ2 ), and observed power ( π).

Regarding the dimensions of LEQ (objective 2), the results showed that coaches evaluate their ideas, principles, and leadership values (i.e., philosophy of leadership) higher than their athletes (*F* = 4.43; *p* = 0.036; *η^2^* = 0.014). In the MSLS dimensions (objective 3), the results showed that coaches assess their concern for the well-being of athletes (that is, support) (*F* = 5.05; *p* = 0.025; *η^2^* = 0.016) and active decision-making higher than their athletes (that is, active management) (*F* = 4.08; *p* = 0.044; *η^2^* = 0.013). On the other hand, coaches evaluate their use of punishment behaviors (ie, negative feedback) (*F* = 6.02; *p* = 0.015; *η^2^* = 0.020) and delay in decision making (ie, passive management) (*F* = 4.95; *p* = 0.027; *η^2^* = 0.016) lower than their athletes. Finally, concerning the LAFQ dimensions (Objective 3), athletes evaluate the team members’ maturity higher than their coaches (*F* = 13.98; *p* <0.001; *η^2^* = 0.044). Regarding the partial eta squared values, all significant differences were of small effect size.

## 4. Discussion

The current study analyzed the coach-athlete relationship in youth football based on the Leadership Efficacy Model. For that, four objectives were established, two of them related to the leadership cycles (objectives 1 and 2), one of them related to the leadership styles (objective 3), and the last one related to the leadership antecedent factors (objective 4). Due to the novelty of studying these goals in youth sports, this study assumed a descriptive and exploratory orientation of analyzing how both the coaches and the athletes from the young National Slovak Leagues perceived the three main factors of the Leadership Efficacy Model.

In the results for Objectives 1 and 2, both athletes and coaches indicated that the leadership philosophy should be increased, suggesting that coaches should express their ideas and leadership principles more explicitly. A leadership philosophy is linked to leadership vision (Gomes, 2020), defined as values, beliefs, assumptions, attitudes, principles, and priorities on the way to future goals. Communicating a positive vision is positively associated with athletes’ individual and team performance (Bormann et al., 2016). A positive and convincing vision increases motivation and performance aspirations and can be relevant in relation to a leadership philosophy. Big data analysis (Park and Seo, 2019) showed that successful sport leaders increase the self-confidence of their athletes, based on scientific analysis and shared visions. They take care of the athletes and present a vision to them. They were characterized by words such as organization, serious consideration, analysis, experience, culture, development, improvement, sharing, decisiveness, positivity, and atmosphere. Unsuccessful leaders were typical for words such as criticism, remarks, incompetence, and insufficiency. They did not care for athletes, had no vision, and failed in communication. The importance of vision for the satisfaction of athletes is demonstrated in literature (Gomes and Resende, 2014), being also important to provide leadership training to help coaches to better articulate their coaching philosophy to the situational demands (Bormann et al., 2016).

It is also important to note that coaches have a better understanding of their leadership, referring to a higher explication of their leadership philosophy than their athletes’ opinions (Gomes et al., 2022). Previous literature has established the importance of coaches’ philosophy in organizing their work with athletes (Martens, 2012). However, our results spread the knowledge on this topic by suggesting that athletes do not attribute to coaches the same high expression of philosophy as coaches do. The results reinforced the importance of clear communication and tools for comprehensibly transferring philosophy into practice and evaluation criteria. This is particularly important because athletes need to understand the link between coach philosophy and subsequent coach behavior. As proposed in the Leadership Efficacy Model, the way to achieve better leadership cycles might be very individual for each coach and team. Coaches should reflect on their beliefs, practices, and criteria to increase their positive impact on athletes (Gomes et al., 2018). Subagyo (2023) expressed the need for each educator to set up the Personal Leadership Philosophy (PLP) Manifesto. PLP manifesto helps educators to reach their goals with students effectively, with authenticity, integrity, and purpose. It should consist of four parts: (1) core beliefs and values; (2) visionary statement; (3) strategic leadership; and (4) leadership commitments and actions. It results from the coach’s reflection on effective leadership styles and self, which results in integrating objective facts and personal values. With that knowledge, the educator/coach can formulate their PLP manifesto. Subagyo (2023) guides the reader on how to follow each step.

Regarding objective 3, the results reinforce the tendency of coaches to better evaluate their behaviors compared to their athletes’ opinions. Coaches believe that they provide greater support and active management and lower negative feedback and passive management than their athletes perceive. Coaches perceive their leadership behavior more positively than their team athletes (Gjesdal et al., 2019; Raimundi et al., 2023). It is worth noting that this pattern was verified in objective 1, where coaches were more optimistic about their tendency to explicitly the leadership philosophy than athletes’ perception. The tendency for coaches to have a more positive perception of their leadership behaviors compared to their athletes can be referred to as a coach-athlete perception gap. One potential explanation for the gap is that coaches may have different expectations and standards for their behavior than their athletes (Myers and Feltz, 2007). Additionally, coaches may be more aware of the effort and intention behind their behaviors. On the contrary, athletes may only perceive the results or consequences of these behaviors. However, it is important to note that this gap can affect the athlete’s development and satisfaction. Thus, coaches must collect feedback from their athletes and strive to bridge this gap to improve their leadership.

The results of objective 4 indicated that the athletes believe that they have better psychological and technical maturity than their coaches perceive. Athletes evaluated their abilities (both at the psychological and technical levels) better than their coaches. This may be due to factors related to athletes’ participation and investment in the team and their personal experiences and perspectives on the team (Almagro et al., 2019). Coaches’ perceptions of their athletes’ abilities and potential can significantly impact athlete motivation and performance (Gjesdal et al., 2019). Coaches who underestimate their athletes’ abilities may inadvertently limit their potential and undermine their confidence. Therefore, coaches must strive for precision and balance in their evaluations of their athletes and communicate their expectations and feedback to promote their athletes’ growth and development.

### 4.1. Practical implications

Rhind et al. (2012) stressed the importance of highly interdependent coach-athlete relationships. Different views on the leadership cycle, style, and antecedents between coaches and athletes can increase the risk of potential individual and team success. The results of our study point out differences between coaches and athletes in how both partners perceived the leadership cycles, styles, and antecedent factors of leadership. Therefore, coaches need to adjust their leadership to the players’ perception of leadership (Gomes, 2020), which have implications for how coaches define the plan of action, apply the plan of action, and define the outcomes. In creating, communicating, and examining these tasks, they can show support, positive feedback, and active management. Although coaches view themselves as effective from the leadership profile point of view, athletes do not perceive them like that (de Albuquerque et al., 2021). As the research of elite coaches from Portugal showed, building a relationship with athletes based on personal respect is one of the four main areas of coaching activity (Gomes et al., 2018). Communicating expectations on the partners and their behavior can help in such misperceptions. Researchers emphasize the need to develop quality interpersonal relationships between coaches and athletes (Jowett, 2017). Underestimating the importance of the coach-athletes relationship can prevent players from developing their full potential (Rhind et al., 2012). Athletes must be taught to think critically and reflect on their performance. They should be willing to ask for feedback from parents, coaches, or teammates to accelerate performance improvement. Coaches should try to be honest with the feedback they give (Ansell and Spencer, 2022). A stronger influence of positive and informational feedback from the coach on the athlete’s perception is necessary for those athletes who emphasize their coach more (Amorose and Nolan-Sellers, 2016). In general, the coach–athlete relationship improves when coaches and athletes perceive athletes’ self-efficacy congruently. More cooperative and effective interactions result from congruence in perceiving high or low efficacy (Stephen et al., 2022).

### 4.2. Future research implications and limitations

When improving relations between coaches and athletes, it is also essential to consider contextual factors, such as individual versus collective sports (Rhind et al., 2012). In team sports, the coach can support the quality of interpersonal relations with players through team-building strategies or by organizing social networking events, for example, end-of-season celebrations. The stress experienced by the coach affects the performance of the athletes and the coach. Training/development of stress-coping strategies in coaches can influence the stress and quality of the coach-athlete relationship (Thelwell et al., 2017). Despite many intervening variables, we can conclude that a coach’s effectiveness depends on quality coach-athlete relationships (Jowett, 2017). Future research can examine the three main factors of the Leadership Efficacy Model (leadership cycles, leadership styles, and antecedent factors of leadership) in stress-coping conditions for both the coaches and athletes, as well as analyze how the interaction between coaches and athletes change along the sport season and affect performance.

We can mention some limitations of the current study. Although we used two different data sources (data from athletes and data from coaches) to avoid common method bias (Rodríguez-Ardura and Meseguer-Artola, 2020), we still conducted a cross-sectional study. Thus, the results of this study cannot claim verified causality. Therefore, the proposed interventions to improve the congruence of leadership perceptions between players and coaches are only recommendatory, based on the results of the current study and the published literature (as referred to above in the discussion). Longitudinal observation/analysis of the specific team/club could bring more information about the possible causal relationships in the Leadership Efficacy Model. Convenient data collection also belongs to the limits of this study. The results cannot be generalized to the population of Slovak football players. To get the results with the possibility of generalization, obtaining a representative sample of a specific team/club/league would be advisable for a complete unit of players and coaches. It would be interesting to examine the relationship with the coaches assistants too. They are in the middle between the athletes and coaches and can also have close relationships with athletes. The study’s results are only valid for male players, and it would be interesting to investigate the perception of coaches’ leadership in female athletes in the future.

## 5. Conclusion

In summary, the results of the current study offer preliminary positive indications of interest in leadership cycles, leadership styles, and antecedent factors of leadership as essential factors to understanding the coach-athlete relationship. Coaches are more positive than athletes when evaluating how they lead athletes and teams. The perception gap between coaches and athletes reinforces the need to develop open communication channels with athletes to adjust both perspectives better. It is also important to note that leadership behavior, having positive perceptions of team members’ maturity, and having a higher perception of good contextual conditions to work, are positively associated with athletes’ perception of performance. The reinforcement of the coach-athlete relationship depends on aligning the perception of both parties, the athletes and the coaches.

## Data availability statement

The raw data supporting the conclusions of this article will be made available by the authors, without undue reservation.

## Ethics statement

The studies involving human participants were reviewed and approved by Ethics Subcommittee for Social and Human Sciences (SECSH), University of Minho. Written informed consent to participate in this study was provided by the participants’ legal guardian/next of kin.

## Author contributions

EL, JS, CM, and AG contributed substantially to the conception or design of the work, the acquisition, analysis, or interpretation of data, drafted the manuscript, and revised it critically and agree to be accountable for all aspects of the work in ensuring that questions related to the accuracy or integrity of any part are appropriately investigated and resolved. All authors contributed to the article and approved the submitted version.

## Conflict of interest

The authors declare that the research was conducted in the absence of any commercial or financial relationships that could be construed as a potential conflict of interest.

## Publisher’s note

All claims expressed in this article are solely those of the authors and do not necessarily represent those of their affiliated organizations, or those of the publisher, the editors and the reviewers. Any product that may be evaluated in this article, or claim that may be made by its manufacturer, is not guaranteed or endorsed by the publisher.

## References

[ref1] Almagro B. J. Sánchez-Alcaraz B. J. López-Sánchez G. F. (2019). Analysis of coaches' and athletes' perceptions of training and competition environment: a comparative study. J. Hum. Kinet. 66, 5–15.30988835

[ref2] Álvarez O. Castillo I. Molina-García V. Tomás I. (2019). Transformational leadership, task-involving climate, and their implications in male junior soccer players: a multilevel approach. Int. J. Environ. Res. Public Health 16:3649. doi: 10.3390/ijerph16193649, PMID: 31569387 PMC6801878

[ref3] Amorose A. J. Nolan-Sellers W. (2016). Testing the moderating effect of the perceived importance of the coach on the relationship between perceived coaching feedback and athletes' perceptions of competence. Int. J. Sports Sci. Coach. 11, 789–798. doi: 10.1177/1747954116676105

[ref4] Ansell D. B. Spencer N. L. I. (2022). "think about what you're doing and why you're doing it": coach feedback, athlete self-regulation, and male youth hockey players. J. Appl. Sport Psychol. 34, 459–478. doi: 10.1080/10413200.2020.1813835

[ref5] Bormann K. C. Schulte-Coerne P. Diebig M. Rowold J. (2016). Athlete characteristics and team competitive performance as moderators for the relationship between coach transformational leadership and athlete performance. J. Sport Exerc. Psychol. 38, 268–281. doi: 10.1123/jsep.2015-0182, PMID: 27385706

[ref6] Burns L. Weissensteiner J. R. Cohen M. Bird S. R. (2022). A survey of elite and pre-elite athletes' perceptions of key support, lifestyle and performance factors. BMC Sports Sci. Med. Rehabil. 14:2. doi: 10.1186/s13102-021-00393-y, PMID: 34980226 PMC8725551

[ref7] Callary B. Werthner P. Trudel P. (2013). Exploring coaching actions based on developed values: a case study of a female hockey coach. Int. J. Lifelong Educ. 32, 209–229. doi: 10.1080/02601370.2012.733974

[ref8] Collins K. (2021). “Coaching philosophy” in Sport coaches' handbook. eds. Gould D. Mallett C. (International Council for Coaching Excellence & Human Kinetics), 31–49.

[ref9] de Albuquerque L. R. Scheeren E. M. Vagetti G. C. de Oliveira V. (2021). Influence of the coach's method and leadership profile on the positive development of young players in team sports. J. Sports Sci. Med. 20, 9–16. doi: 10.52082/jssm.2021.9, PMID: 33707981 PMC7919346

[ref10] Gjesdal S. Stenling A. Solstad B. E. Ommundsen Y. (2019). A study of coach-team perceptual distance concerning the coach-created motivational climate in youth sport. Scand. J. Med. Sci. Sports 29, 132–143. doi: 10.1111/sms.13306, PMID: 30230049

[ref11] Gomes A. R. (2020). “Coaching efficacy: the leadership efficacy model” in Coaching for human development and performance in sports. eds. Resende V. R. Gomes A. R. (Springer), 43–72.

[ref12] Gomes A. R. Almeida A. Resende R. (2020). Athletes' perception of leadership according to their perceptions of goal achievement and sport results. Percept. Mot. Skills 127, 415–431. doi: 10.1177/0031512519892384, PMID: 31840557

[ref13] Gomes A. R. Araújo V. Resende R. Ramalho V. (2018). Leadership of elite coaches: the relationship among philosophy, practice, and effectiveness criteria. Int. J. Sports Sci. Coach. 13, 1120–1133. doi: 10.1177/1747954118796362

[ref14] Gomes A. R. Gonçalves A. Morais C. Simães C. Resende R. (2022). Leadership efficacy in youth football: Athletes' and coaches' perspectives. Int. Sport Coach. J. 9, 170–178. doi: 10.1123/iscj.2020-0128

[ref15] Gomes A. R. Resende R. (2014). “Assessing leadership styles of coaches and testing the augmentation effect in sport” in Contemporary topics and trends in the psychology of sports (Nova Science Publisher), 115–137.

[ref16] Gomes A. R. Simães C. Morais C. Resende R. (2021). Psychometric properties of the multidimensional sport leadership scale comparison to multifactorial leadership questionnaire. Int. J. Sport Psychol. 52, 189–212. doi: 10.7352/IJSP.2021.52.189

[ref17] Gould D. Pierce S. Cowburn I. Driska A. (2017). How coaching philosophy drives coaching action: a case study of renowned wrestling coach J Robinson. Int. Sport Coach. J. 4, 13–37. doi: 10.1123/iscj.2016-0052

[ref18] Hodge K. Lonsdale C. Jackson S. A. (2009). Athlete engagement in elite sport: an exploratory investigation of antecedents and consequences. Sport Psychol. 23, 186–202. doi: 10.1123/tsp.23.2.186

[ref19] Jowett S. (2017). Coaching effectiveness: the coach–athlete relationship at its heart. Curr. Opin. Psychol. 16, 154–158. doi: 10.1016/j.copsyc.2017.05.006 28813341

[ref1002] Kim J. Panza M. Evans M. B. (2021). Group dynamics in sport. In Zenko Z. Jones L. (Eds.). Essentials of exercise and sport psychology: An open access textbook. (Hayward, CA: Society for Transparency, Openness, and Replication in Kinesiology). 613–642.

[ref20] Mageau G. A. Vallerand R. J. (2003). The coach-athlete relationship: a motivational model. J. Sports Sci. 21, 883–904. doi: 10.1080/0264041031000140374 14626368

[ref21] Martens R. (2012). “Successful coaching” in Human Kinetics

[ref1003] McGuckin M. E. Turnnidge J. Bruner M. Lefebvre J. S. Côté J. (2022). Exploring youth sport coaches’ perceptions of intended outcomes of leadership behaviours. Int. J. Spor. Sci. Coac. 17, 463–476. doi: 10.1177/17479541221076247 PMC13029527 41908295

[ref22] Myers N. D. Feltz D. L. (2007). “The coach–athlete relationship: a tripartite efficacy perspective” in Handbook of sport psychology. eds. Tenenbaum G. Eklund R. C.. 3rd ed (Wiley), 443–460.

[ref23] Park E.-M. Seo J.-H. (2019). A study on leadership typology in sports leaders based on big data analysis. J. Korea Convergence Soc. 10, 191–198. doi: 10.15207/JKCS.2019.10.7.191

[ref24] Raimundi M. J. Corti J. F. Pérez-Gaido M. Alvarez O. Castillo I. (2023). Which assessment of coach-created motivational climate better predicts young Athletes' engagement over a season? Athletes' perceptions and match observations do. Sustainability 15:5179. doi: 10.3390/su15065179

[ref25] Reardon I. P. Van Raalte J. L. Brewer B. W. DeSousa M. (2022). Effects of autonomy supportive behaviors and season outcome on Athletes' perceptions of coaches. Int. J. Coach. Sci. 16, 3–24.

[ref26] Reinboth M. Duda J. L. (2006). Perceived motivational climate, need satisfaction and indices of well-being in team sports: a longitudinal perspective. Psychol. Sport Exerc. 7, 269–286. doi: 10.1016/j.psychsport.2005.06.002

[ref27] Rhind D. J. A. Jowett S. Yang S. X. (2012). A comparison of athletes' perceptions of the coach-athlete relationship in team and individual sports. J. Sport Behav. 35, 433–452.

[ref28] Rodríguez-Ardura I. Meseguer-Artola A. (2020). Editorial: how to prevent, detect and control common method variance in electronic commerce research. J. Theor. Appl. Electron. Commer. Res. 15. doi: 10.4067/S0718-18762020000200101

[ref29] Stephen S. A. Habeeb C. M. Arthur C. A. (2022). Congruence of efficacy beliefs on the coach-athlete relationship and athlete anxiety: athlete self-efficacy and coach estimation of athlete self-efficacy. Psychol. Sport Exerc. 58:102062. doi: 10.1016/j.psychsport.2021.102062

[ref30] Subagyo M. (2023). Crafting an effective leadership philosophy manifesto for education settings. Available at: https://ssrn.com/abstract=4467756 (Accessed June 2, 2023)

[ref31] Thelwell R. C. Wagstaff C. R. D. Chapman M. T. Kenttä G. (2017). Examining coaches' perceptions of how their stress influences the coach-athlete relationship. J. Sports Sci. 35, 1928–1939. doi: 10.1080/02640414.2016.1241422, PMID: 27719269

[ref32] Vallée C. N. Bloom G. A. (2016). Four keys to building a championship culture. Int. Sport Coach. J. 3, 170–177. doi: 10.1123/iscj.2016-0010

[ref33] Warmath D. Winterstein A. P. Myrden S. (2022). Parents and coaches as transformational leaders: motivating high school athletes' intentions to report concussion symptoms across socioeconomic statuses. Soc. Sci. Med. 292:114559. doi: 10.1016/j.socscimed.2021.114559, PMID: 34776287

